# A Straight Talking Introduction to Psychiatric Diagnosis

**DOI:** 10.1192/pb.bp.114.050369

**Published:** 2016-04

**Authors:** Brian Martindale

The lead up to the publication of DSM-5 was accompanied by a great deal of debate and argument within psychiatry. The director of the American National Institute of Mental Health (NIMH) stated of current diagnostic systems that ‘Patients … deserve better … the weakness is [the systems’] lack of validity’ and that NIMH would be re-orienting its research away from DSM categories.

As this excellent pocket book makes clear, giving a psychiatric diagnosis, whatever its usefulness, may have serious consequences. The book is one of a series that highlights alternative perspectives on mental health problems. It is written not only for psychologists and people with personal experience of mental disorder, but also for psychiatrists.

There are many psychiatrists who genuinely think about their ‘patients’ in psychosocial, biological and developmental terms, but there is much research evidence referred to in this book which supports my own experience that, sadly, much of psychiatric thinking and practice sees mental illness as essentially organic. The author herself equates the medical model with an organic one, rather than understanding that a medical model that does not pay attention to psychosocial factors is very limited.

Nevertheless, the book is concise, well written and with full references. Johnstone states the arguments clearly and lays out short chapters giving a well-balanced mix of theory, research evidence and personal experiences. She gives an introduction to an alternative to diagnosis: that of building formulations with patients. She gives sound reasons why she prefers formulation as an alternative, rather than an addition, to diagnosis. I agree with her that there can be a risk to many patients in giving a diagnosis, but there can also be benefits.

The book should be useful in the training of all psychiatrists, for continuing professional development (CPD) and for team development.

